# The C-Terminal Fragment of Prostate-Specific Antigen, a 2331 Da Peptide, as a New Urinary Pathognomonic Biomarker Candidate for Diagnosing Prostate Cancer

**DOI:** 10.1371/journal.pone.0107234

**Published:** 2014-09-18

**Authors:** Kenji Nakayama, Takahiro Inoue, Sadanori Sekiya, Naoki Terada, Yu Miyazaki, Takayuki Goto, Shigeki Kajihara, Shin-Ichiro Kawabata, Shinichi Iwamoto, Kuniko Ikawa, Junko Oosaga, Hiroaki Tsuji, Koichi Tanaka, Osamu Ogawa

**Affiliations:** 1 Department of Urology, Graduate School of Medicine, Kyoto University, Sakyo-ku, Kyoto, Japan; 2 Koichi Tanaka Laboratory of Advanced Science and Technology, Shimadzu Corporation, Nakagyou-ku, Kyoto, Japan; 3 Department of Clinical Laboratory, Kyoto University Hospital, Sakyo-ku, Kyoto, Japan; Innsbruck Medical University, Austria

## Abstract

**Background and Objectives:**

Prostate cancer (PCa) is one of the most common cancers and leading cause of cancer-related deaths in men. Mass screening has been carried out since the 1990s using prostate-specific antigen (PSA) levels in the serum as a PCa biomarker. However, although PSA is an excellent organ-specific marker, it is not a cancer-specific marker. Therefore, the aim of this study was to discover new biomarkers for the diagnosis of PCa.

**Materials and Methods:**

We focused on urine samples voided following prostate massage (digital rectal examination [DRE]) and conducted a peptidomic analysis of these samples using matrix-assisted laser desorption/ionization time-of-flight mass spectrometry (MALDI-TOF/MS^n^). Urinary biomaterials were concentrated and desalted using CM-Sepharose prior to the following analyses being performed by MALDI-TOF/MS^n^: 1) differential analyses of mass spectra; 2) determination of amino acid sequences; and 3) quantitative analyses using a stable isotope-labeled internal standard.

**Results:**

Multivariate analysis of the MALDI-TOF/MS mass spectra of urinary extracts revealed a 2331 Da peptide in urine samples following DRE. This peptide was identified as a C-terminal PSA fragment composed of 19 amino acid residues. Moreover, quantitative analysis of the relationship between isotope-labeled synthetic and intact peptides using MALDI-TOF/MS revealed that this peptide may be a new pathognomonic biomarker candidate that can differentiate PCa patients from non-cancer subjects.

**Conclusion:**

The results of the present study indicate that the 2331 Da peptide fragment of PSA may become a new pathognomonic biomarker for the diagnosis of PCa. A further large-scale investigation is currently underway to assess the possibility of using this peptide in the early detection of PCa.

## Introduction

Prostate cancer (PCa) is one of the most common cancers and the leading cause of cancer-related deaths in men [1]. The mechanisms underlying the development of PCa have not yet been determined because of its clinical and histological heterogeneity. The incidence for PCa has markedly increased in Japan recently [2], [3]. The large-scale clinical detection of prostate-specific antigen (PSA) levels in the serum as a PCa biomarker has been carried out since the 1990s [3]–[7]. Although the overall benefits and risks of population PSA screening for prostate cancer continue to be assessed [8], PSA is known to be an excellent organ-specific, but not a cancer-specific marker [9], which continues to be a clinical problem. This is further compounded by the longer-living, aging population and elevated PSA levels associated with increasing age [3], [10].

Although the sensitivity of PSA in the detection of cancer is high, its specificity is limited, and screening healthy men often causes false cancer alarms (e.g. due to inflammation or benign hyperplasia) and unnecessary prostate biopsies [3], [11]. Several issues have been identified regarding the sub-optimal sensitivity of PSA testing for PCa screening, which lead to unnecessary biopsies, overdiagnoses, and overtreatments [12]–[17]. A significant amount of effort in research is currently being directed towards improving the accuracy of PCa screening [12]. Moreover, a strong emphasis has been placed on the need to identify novel biomarkers for the diagnosis of PCa.

Proteomic techniques applied to serum, plasma, and urine may provide valuable information regarding biomarkers and marker patterns, which may be used to improve the detection of cancer [18]. In the present study, we focused on urine samples voided following prostate massage (digital rectal examination [DRE]), which were expected to contain many peptides and protein fragments secreted from prostatic microenvironments that could enable the detection of secreted prostate products as potential sources of PCa-specific biomarkers [18], [19]. Therefore, we conducted peptidomic and proteomic analyses of urine samples using matrix-assisted laser desorption/ionization time-of-flight mass spectrometry (MALDI-TOF/MS^n^) in order to discover new potential pathognomonic biomarker candidates for the diagnosis of PCa.

## Materials and Methods

### Ethics Statement

This study was conducted with the approval of the Ethics Committee of the Kyoto University Graduate School of Medicine. Informed consent was obtained from all cases for the examinations and experiments conducted. Clinical materials were used after written informed consent was obtained, according to protocols approved by the Institutional Review Board of Kyoto University Hospital.

### Patients

The individuals from which urine samples were collected following prostate massage (digital rectal examination [DRE]) were classified into 2 groups; i.e., PCa patients and non-cancer subjects. The confirmatory diagnosis of PCa was made by a histological diagnosis from prostate biopsy specimens or prostate glands removed following surgery, when performed. Fifty samples were collected from PCa patients, and their clinical characteristics were shown in Table 1. The clinical characteristics of non-cancer subjects were shown in Table 2. All urine samples from the non-cancer group were collected prior to holmium laser enucleation of the prostate (HoLEP), transurethral resection of the prostate (TURP), and needle biopsy of the prostate gland, when performed. The diagnoses of non-cancer subjects were defined as non-malignant by histological diagnoses of prostate glands removed by HoLEP and TURP and prostate specimens obtained by needle biopsy, except two cases (No. 14 and 15) who did not receive needle biopsy because of very low serum PSA and normal DRE. Nineteen non-cancer samples were collected. All histological diagnoses were confirmed by genitourinary pathologists in our hospital.

**Table 1:** Clinical characteristics of PCa patients.

PCa No.	Age(years old)	Serum PSA(ng/mL)	Clinical stage	Bioptic Gleason Score (bGS)
**1** a	64	4.23	cT1cN0M0	3+3
**2** a	64	4.50	cT2aN0M0	3+4
**3** a	66	4.66	cT1cN0M0	4+3
**4** a	65	4.89	cT1cN0M0	4+3
**5** a	65	5.92	cT1cN0M0	4+3
**6** a	72	6.15	cT1cN0M0	3+4
**7** a	74	6.43	cT2cN0M0	3+3
**8** a	65	7.25	cT2aN0M0	3+3
**9** a	64	7.46	cT2aN0M0	3+3
**10** a	63	9.06	cT2aN0M0	3+3
**11** a	65	9.56	cT1cN0M0	4+4
**12** a	67	10.5	cT1cN0M0	3+3
**13** a	65	11.7	cT2aN0M0	3+3
**14** a	64	18.8	cT2aN0M0	3+3
**15** a	66	66.0	cT2bN0M0	4+3
**16**	73	3.82	cT2aN0M0	3+4
**17**	67	4.05	cT1cN0M0	3+4
**18**	80	4.27	*N/A*	3+4
**19**	60	4.29	cT2aN0M0	4+3
**20**	71	4.33	cT2aN0M0	3+4
**21**	68	4.54	cT2aN0M0	4+4
**22**	74	4.66	cT2aN0M0	3+4
**23**	47	4.92	cT2aN0M0	3+4
**24**	57	4.98	cT1cN0M0	4+4
**25**	68	5.38	cT1cN0M0	3+3
**26** b	74	5.40	pT1aN0M0	*N/A*
**27**	68	5.44	cT2aN0M0	3+3
**28**	65	5.72	cT1cN0M0	4+3
**29**	61	6.51	cT1cN0M0	4+4
**30**	50	6.79	cT1cN0M0	3+4
**31**	74	7.38	cT2cN0M0	3+3
**32**	71	7.51	cT1cN0M0	4+4
**33**	69	7.61	cT2aN0M0	4+3
**34**	51	7.70	cT1cN0M0	3+4
**35**	65	7.93	cT1cN0M0	3+3
**36**	61	8.65	cT1cN0M0	3+3
**37**	75	9.39	cT1cN0M0	3+4
**38**	67	10.0	cT2aN0M0	4+3
**39**	60	10.6	cT2aN0M0	4+4
**40**	66	11.1	cT1cN0M0	4+4
**41**	69	12.1	cT2aN0M0	3+4
**42**	69	14.6	cT2aN0M1	4+4
**43**	77	14.9	cT1cN0M0	4+3
**44**	63	18.8	cT2aN0M0	3+3
**45**	85	30.9	cT3aN0M0	4+3
**46**	74	33.3	cT3aN0M0	4+4
**47**	80	33.5	cT3aN0M0	4+4
**48**	88	2261	cT3aN1M1b	3+4
**49**	76	3242	cT3aN1M1b	4+5
**50**	78	8802	cT4N1M1b	4+4

a These data were utilized as a discovery set.

b This patient was diagnosed with PCa after HoLEP for BPH.

**Table 2:** Clinical characteristics of non-cancer subjects.

No.	Age(years old)	Serum PSA (ng/mL)	Surgical procedure
**1** a	63	1.00	HoLEP
**2** a	72	3.19	HoLEP
**3** a	73	3.21	HoLEP
**4** a	75	3.32	HoLEP
**5** a	70	5.03	HoLEP
**6** a	72	6.16	HoLEP
**7** a	64	6.29	HoLEP
**8** a	72	8.51	HoLEP
**9** a	79	9.23	HoLEP
**10** a	64	13.0	HoLEP
**11** a	63	14.6	HoLEP
**12** a	65	19.2	HoLEP
**13**	72	3.81	TURP
**14**	84	0.85	Non
**15**	49	1.85	Non
**16**	71	5.83	Needle biopsy
**17**	78	6.28	Needle biopsy
**18**	75	9.47	Needle biopsy
**19**	51	22.4	Needle biopsy

a These data were utilized as a discovery set.

### Chemicals and Reagents

Tris [hydroxymethyl] aminomethane (Tris), Triton X-100, and urea were purchased from Sigma-Aldrich (St Louis, MO, USA). Molecular biology grade 3-[(cholamidopropyl) dimethylammonio]-1-propanesulfonate (CHAPS) was purchased from Calbiochem (San Diego, CA, USA). All water used in this experiment, except for the HPLC system, was prepared using a Milli-Q system (Millipore, Bedford, MA, USA). Acetonitrile (ACN) with and without 0.1% formic acid (FA), and water with and without 0.1% FA for LC-MS Chromasolv were purchased from Sigma-Aldrich (St Louis, MO, USA). All other reagents were of the highest quality commercially available.

### Urine sample collection

Urine samples were collected as follows: A urologist performed DRE by gently massaging each side of the prostate gland three times, which stimulated the movement and release of prostate fluids into the urethra. These prostate fluids were collected when the subjects voided urine following the massage. The urine samples collected were filtered with 100-µm nylon cell strainers (BD Falcon, San Jose, CA, USA) and centrifuged at 2,000×g for 10 min at 20°C to exclude urinary debris. After centrifugation, the supernatants were filtered again with the cell strainers. The filtered supernatant of the urine samples was stored at −80°C until later analyses.

### Urine sample preparation

In the present study, urinary peptides and protein fragments captured by ion-exchange Sepharose resin were analyzed using MALDI-TOF/MS^n^. CM-Sepharose Fast Flow (GE Healthcare, Buckinghamshire, UK) was used as an ion-exchange resin to capture and concentrate the peptides and protein fragments in the urine sample, as well as to desalt concentrated samples. An in-house built MALDI digital ion trap (DIT) TOF/MS^n^ manufactured by Shimadzu Corporation (Kyoto, Japan) was utilized for mass spectrum analysis. Differential analyses of urinary mass spectrum data between 2 subject groups (non-cancer and PCa) were performed using the ion-trap reflectron mode of MALDI-DIT-TOF/MS. Peak identifications were also performed in the reflectron ion-trap mode. Peak lists obtained from MS^2^ spectra were used to identify the amino acid sequence with the Mascot MS^2^ search engine (Matrix science, London, UK).

Frozen urine samples (1.0 mL) were thawed by centrifugation at 1,000×g for 15 min at 20°C. Four hundred µL of lysis buffer (9 M urea, 2% CHAPS, 50 mM Tris, pH 9) was added to each urine sample and the mixtures were incubated for 30 min at 4°C with rotation. The denatured urine samples were added to 3.6 mL binding buffers (100 mM ammonium acetate, pH 4, 0.05% Triton-X). Ninety µL of 30% activated CM-Sepharose was added to each of the above binding buffer mixtures and vortexed well. The tubes were incubated for 30 min at 4°C with rotation. After being incubated, the tubes were centrifuged at 1,000×g for 10 min at 20°C. The supernatants were discarded using a 5-mL Finnpipette F1 (Thermo Fisher Scientific, Vantaa, Finland). The precipitates, CM-Sepharose-captured peptides and protein fragments, were transferred into 1.5-mL microtubes.

The resins were washed three times with binding buffer and distilled water for LC-MS. Washing involved centrifuging (3,000×g for 3 min at 20°C with binding buffer and 5,000×g for 3 min at 20°C with distilled water), discarding the supernatants, and adding washing solutions. In the final washing step, the tubes were centrifuged at 10,000×g for 5 min at 20°C and as much washing distilled water as possible was withdrawn. Twenty-five µL of 1% trifluoroacetic acid (TFA) solution was added to the CM-Sepharose resin to elute the captured peptides and protein fragments, and pipetted well for 2 min. The tubes were centrifuged at 12,000×g for 5 min at 20°C. Only the supernatants were withdrawn and transferred to other microtubes. The tubes were centrifuged at 14,000×g for 5 min at 20°C and the supernatants were transferred to other microtubes. These supernatants served as the urine sample extracts.

We targeted intact peptides and protein fragments between 1000 and 5000 Da in the urine samples because their amino acid sequences could be directly confirmed by MALDI-DIT-TOF/MS^2^ in this molecular weight range.

### DHB matrix and MALDI plate

DHB (2,5-dihydroxybenzoic acid) matrix solution was prepared by dissolving 5 mg of DHB (LaserBio Labs, Sophia-Antipolis Cedex, France) in 500 µL of 50% acetonitrile/0.1% TFA. In the DHB matrix analysis, a 0.5 µL aliquot of the eluate was mixed with an equal volume of DHB matrix solution. The premixed samples were spotted onto the following plates and air-dried at room temperature; µFocus MALDI plate 900-µm (384 circles, HST, Inc., Newark, NJ, USA) for the differential analyses of mass spectra or a 384-position stainless steel target plate (Shimadzu Biotech, Kyoto, Japan) for quantitative analyses of a potential biomarker for diagnosis the of PCa.

### Mass Spectrometric Measurements

In MALDI-DIT-TOF/MS^n^, argon gas was used for collision-induced dissociation fragmentation, and helium gas was used for ion cooling. MS and MS^n^ analyses were carried out in the positive ion extraction mode. All analyses were performed under a high vacuum condition (5×10^−5^ Pa or less).

Mass spectra were externally calibrated with a manually prepared 7-peptide standard consisting of angiotensin II ([M+H]^+^1046.54), angiotensin I ([M+H]^+^1296.69), Glu-fibrinopeptide B ([M+H]^+^1570.68), N-acetyl renin substrate ([M+H]^+^1800.94), ACTH (1–17) ([M+H]^+^2093.09), ACTH (18–39) ([M+H]^+^2465.20), and ACTH (7–38) ([M+H]^+^3657.93).

Measurement conditions in the differential analyses of mass spectra were fixed as follows: 1) a µFocus plate was used as the target plate, 2) the laser irradiation-positions were manually focused to the sample-matrix-mixture of each well, 3) the number of irradiation points was 100, 4) the number of irradiation shots was 64, and 5) the laser power was fixed at 85 arbitrary units. A total of 6400 spectra were measured per well. Analyses were performed in duplicate and an extracted aliquot was placed into 2 wells. Therefore, the data of one urine sample were composed of 4 raw data points with 6400 spectra. The coefficient of variation concerning intensities at several major peaks, especially at a biomarker candidate peak, became less than 10% under these analytical conditions (data not shown).

### Data processing of MS spectrum data and multivariate analysis

Mass spectrum raw data were exported through DIT-FP Console software (Shimadzu Corporation, Kyoto, Japan) for MALDI-DIT-TOF/MS as mzXML files and imported into MATLAB software version 7.11 (The Mathworks Inc., Natick, MA, USA). MATLAB was supported by Bioinformatics Toolbox and Statistics Toolbox. The following data processing was performed using the toolbox functions in order to detect and align peaks from multiple spectra. 1) A smooth spectrum was obtained using locally weighted scatter plot smoothing (mslowess function [20]). 2) The baseline was subtracted from the spectrum using a spline approximation (msbackadj function [21]). 3) The spectrum was normalized by dividing by the total ion current. 4) Peaks were detected by filtering noise with the undecimated discrete wavelet transform (mspeaks function [22]). 5) Peak lists were generated from multiple spectra by repeating processing from 1) to 4). 6) Peak lists were aligned to match peaks across spectra (mspalign function [23]). CSV-formatted peak lists were then exported from MATLAB and imported into a multivariate analysis software, i.e., SIMCA 13.0.3 (Umetrics AB, Umeå, Sweden; [24], [25]). All imported data were Pareto scaled prior to multivariate analysis. Principal component analysis (PCA) was used for unsupervised multivariate analyses, and orthogonal partial least squares discriminant analysis (OPLS-DA) was used for supervised multivariate analyses. PCA and OPLS-DA models were depicted as score plots (principal component [PC1, PC2]), which displayed any intrinsic grouping of samples based on spectral variations. In the OPLS-DA analysis, potential biomarkers were selected based on the S-plot. The plot of the covariance versus the correlation in conjunction with the variable trend plots resulted in the easier visualization of data. The variables that changed most were plotted at the top or bottom of the “S” shaped plot, and those that did not vary significantly were plotted in the middle.

### Peptide purification and Edman degradation

In the Edman degradation analysis of the N-terminal amino acid sequence of the *m/z* 2332 peptide, purification of the peptide by reverse-phase HPLC was performed using a Shimadzu LC-20AD series HPLC system equipped with a SPD-M20A diode array detector (Shimadzu Corporation, Kyoto, Japan). A Luna C-18 column (5 µm particle size; 4.6 mm ID×250 mm; Phenomenex, Torrance, CA, USA) was utilized for the purification process. The HPLC conditions used were as follows: the column oven temperature was 40°C, the flow rate was 1.0 mL/min, water with 0.1% FA and ACN with 0.1% FA were used as solvents A and B, respectively, the gradient condition was maintained at 10%, as solvent B, for 2 min after a 20-µL sample injection and was linear from 10% to 80% for 15 min. Eluted peptides were monitored at 276 nm. The peptide eluted between 6.6 min and 6.9 min was pooled into a microtube.

The automated Edman degradation of the N-terminal amino acid sequence was performed on RP-HPLC purified peptides using a Procise Model 491 sequencer (Applied Biosystems, Inc., Foster City, CA, USA).

### AQUA peptides and quantitative analyses

We obtained non-labeled synthetic peptides and stable isotope-labeled synthetic peptides from Sigma-Aldrich Japan (Ishikari, Japan). A custom AQUA peptide set for mass spectrometry was synthesized by Sigma-Aldrich Japan to greater than 95% purity, was portioned in 1.0 nmol, and was stored as lyophilized powder at −30°C until use. The amino acid sequence of the AQUA isotope-labeled peptide was YTKVVHY[R-^13^C_6_, -^15^N_4_]KWIKDT[I-^13^C_6_, -^15^N]VANP. Subsequently, the molecular weight increment in the isotope-labeled peptide (relative to the non-labeled peptide) was 17 Da. This peptide was utilized as an internal standard (iSTD) for the quantitative analyses of a potential biomarker candidate for the diagnosis of PCa. The AQUA peptides were dissolved to 1.0 pmol/µL using 1000 µL 10% (v/v) acetic acid of LC-MS grade (Sigma-Aldrich, Saint Louis, MO, USA). The stock solution was (100 µL) was placed in 500 µL microtubes and was stored in −30°C until use.

Twenty pmoles (20 pmol) of the iSTD peptide (20 µL solution) was spiked into the mixture solution of the urine sample and lysis buffer, and then incubated for 30 min at 4°C with rotation. The following procedures were performed according to the urine sample preparation method. Quantitative analyses were performed using MALDI-DIT-TOF/MS. In quantitative analyses using MALDI-DIT-TOF/MS, the measurement conditions were fixed as follows: 1) a stainless steel target plate was used, 2) the laser irradiation positions were manually focused to the sample-matrix-mixture of each well, 3) the number of irradiation points was 200, 4) the number of irradiation shots was 16, and 5) laser power was fixed at 85 arbitrary units. A total of 3200 spectra were measured per well. Analyses were performed in duplicate and an extracted aliquot was placed into 2 wells. Therefore, the data of one urine sample were composed of 12800 spectra. This method was referred to and modified from the methods reported by Sogawa K., et al. [26] and Tyan YC., et al. [27]. The concentrations of the biomarker candidate were calculated on the basis of the ratio of the peak-area at *m/z* 2332 against that at *m/z* 2349.

### Statistics

All statistical analyses in the present study were performed using JMP 10.0.2 statistical software (SAS Institute Japan Inc., Tokyo, Japan). The Mann–Whitney–Wilcoxon, also called the Mann–Whitney U, test was employed to identify a non-parametric significant difference between two groups. Differences with *p* values of less than 0.05 were considered to be significant. Receiver operating characteristic (ROC) curves estimated by the statistical software were used to assess the accuracy of diagnostic tests that yielded continuous test results. Moreover, ROC curves were utilized to assess the predictive power of the pathognomonic biomarker identified in the present study [28].

## Results

### Age and serum PSA levels are not significantly different between non-cancer subjects and PCa patients used as a discovery set

**Figure 1:**
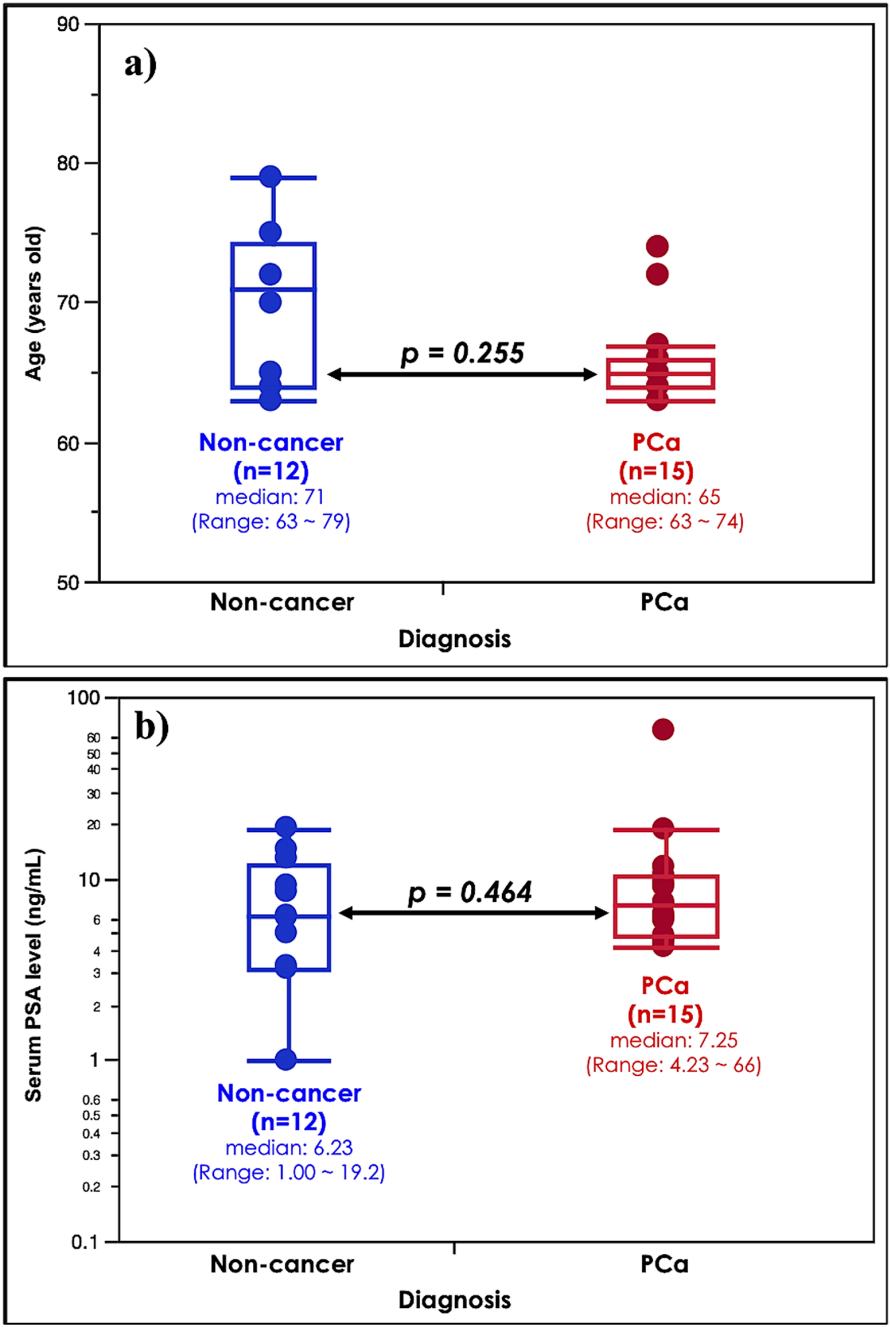
Clinical characteristics of non-cancer subjects and prostate cancer patients used as a discovery set. Age (a) and serum PSA (b) distributions between non-cancer subjects and prostate cancer (PCa) patients were shown. No significant differences were observed in age or serum PSA between non-cancer subjects (n = 12) and PCa patients (n = 15). Their *p* values were 0.255 and 0.464, respectively.

In order to assess mass spectrum differences between the urine samples of 12 non-cancer subjects and 15 PCa patients, the following analyses were performed as a discovery set. As shown in Figure 1a and b, no significant differences were observed in the age or serum PSA distributions between the non-cancer and PCa groups. These results indicated that age-matched urine samples could be used in subsequent analyses.

### Mass spectra using MALDI-DIT-TOF/MS analyses

**Figure 2:**
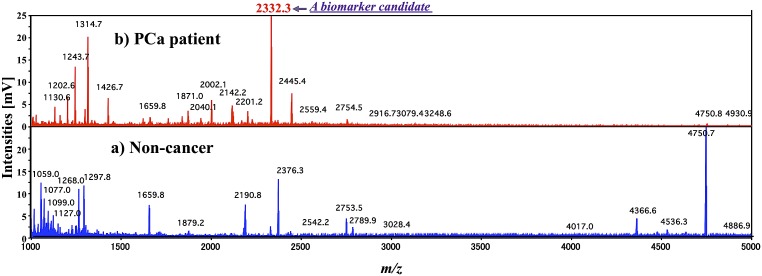
Typical mass spectra of urinary peptides and protein fragments analyzed using the reflectron ion-trap mode of MALDI-DIT-TOF/MS. After concentrating and desalting urine samples by CM-Sepharose, the eluent was extracted with 1.0% TFA solution. DHB was used as the MALDI matrix. Figure 2a and 2b show the analytical results of urine extracts from non-cancer subjects and PCa patients, respectively.

High-resolution and high–sensitivity MALDI-DIT-TOF/MS^n^ was utilized to analyze urinary peptides and protein fragments in the present study. The typical mass spectra of the urinary peptides and protein fragments analyzed using the ion-trap reflectron mode of MALDI-DIT-TOF/MS are shown in Figure 2. After the concentration and desalting of urine samples by CM-Sepharose, the eluent was extracted with 1.0% TFA solution. DHB was used as the MALDI matrix. The mass spectra of extracted samples from a) non-cancer subjects and b) PCa patients were compared. Several differences were detected in these mass spectra between the two groups. The most pronounced peak was detected at *m/z* 2332.3.

### Multivariate analyses of the mass spectra of urinary extracts using SIMCA software

The exported peak-matrix files by MATLAB were imported into a multivariate analysis software, i.e., SIMCA 13.0.3. All imported data were Pareto scaled for multivariate analyses. A principal components analysis (PCA) was initially conducted on the spectra of urine samples to determine whether mass spectrum differences existed between the non-cancer and PCa groups. An orthogonal partial least square discriminant analysis (OPLS-DA) was then used to maximize covariance between the measured data (peak positions and peak intensities) and disease classification. An S-plot was used to select and indicate the potential biomarker candidates of significant biomaterials related to the group separation.

**Figure 3:**
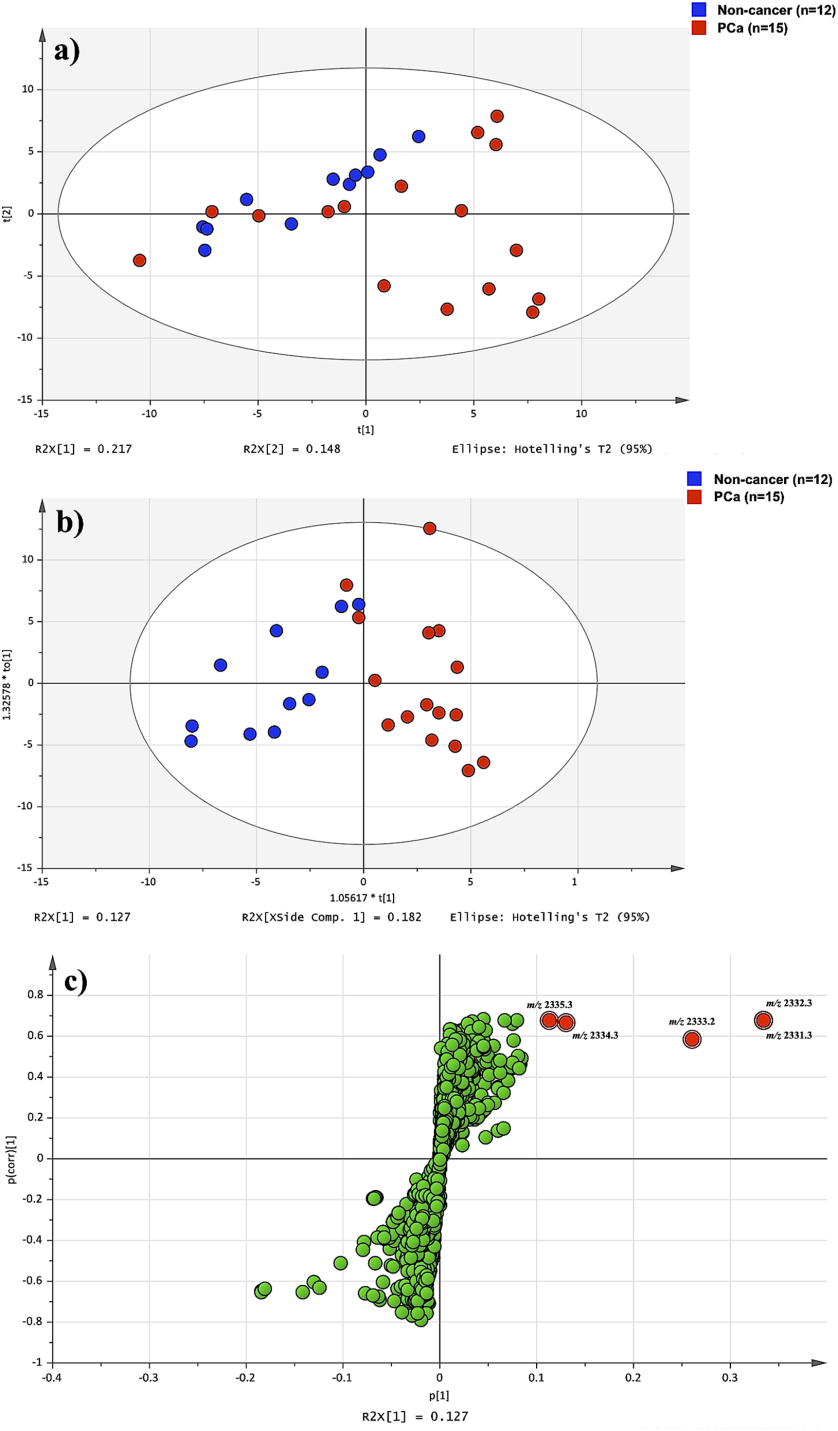
Multivariate analyses of the mass spectra of extracts from the urine samples of non-cancer subjects and PCa patients. (a) PCA score plot indicating the discrimination between non-cancer and PCa in the discovery set. (b) OPLS-DA score plot showing a clear discrimination between non-cancer and PCa. (c) S-plot corresponding to the OPLS-DA score plot shown in Figure 3b. A potential urinary biomarker candidate was extracted at a peak around *m/z* 2332.3 for the diagnosis of PCa patients.

The PCA score plots in PC1 (R^2^ = 0.217) and PC2 (R^2^ = 0.148) showed unclear clusters between the non-cancer and PCa groups (R^2^X[cum]: 0.556 and Q^2^[cum]: 0.271; Figure 3a). In contrast, the OPLS-DA score plots distinguished the PCa group from the non-cancer group (R^2^X[cum]: 0.309, R^2^Y[cum]: 0.722 and Q^2^[cum]: 0.058; Figure 3b). The OPLS-DA was an adequate model for distinguishing the PCa group from the non-cancer group. An S-plot analysis based on the OPLS-DA model relative to the proportion of the peak intensities of mass spectra concerning PCa and non-cancer is shown in Figure 3c. The peak intensities around *m/z* 2332.3 were isolated from the S-plot as new potential pathognomonic biomarkers for distinguishing between the non-cancer and PCa groups.

### Identification of peptide fragments at m/z 2332.3

**Figure 4:**
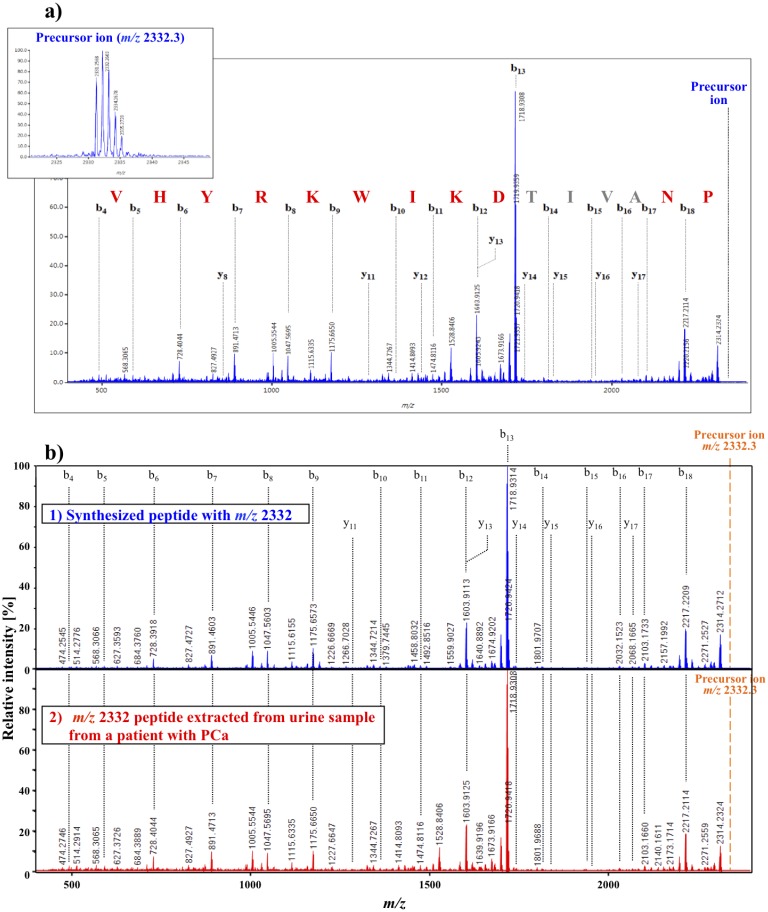
A typical mass spectrum after MS^2^ analysis of the peptide with *m/z* 2332 using MALDI-DIT-TOF/MS^2^. (a) The amino acid sequence of *m/z* 2332 was identified in the urine samples of PCa patients and estimated using MS^2^ analyses and the Mascot MS^2^ search engine. The N-terminal amino acid sequences of the peptide were confirmed completely based on data obtained by the Edman degradation of the purified peptide. The sequence was confirmed as follows: YTKVVHYRKWIKDTIVANP. (b) MS^2^ spectrum comparisons of the *m/z* 2332 peptide between a synthesized material (1) and extracted biomaterial from the urine samples of PCa patients (2). The resulting 2 MS^2^ spectra of the *m/z* 2332 peptides matched completely.

**Figure 5:**
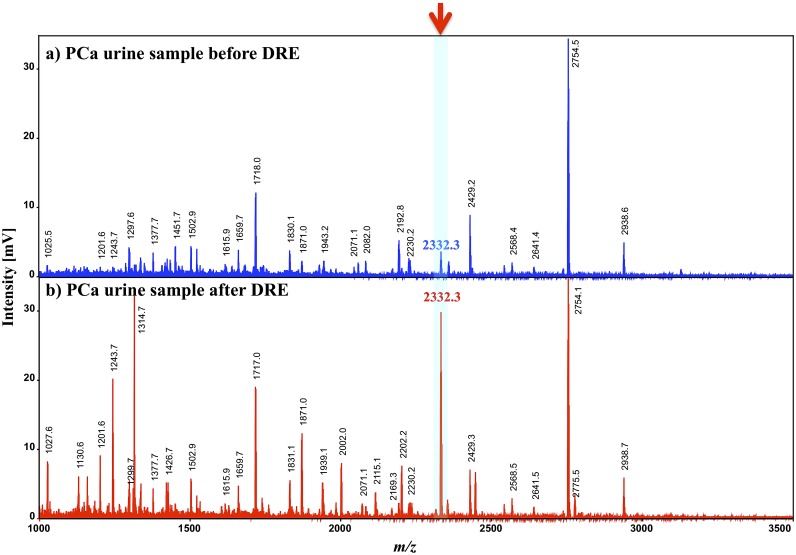
Mass spectrum comparison of the PCa urinary samples before (a) and after (b) DRE.

The mass spectrum of a urine sample from a PCa patient by MALDI-DIT-TOF/MS^2^ analysis is shown in Figure 4a. The Mascot MS^2^ search engine of the MS^2^ mass spectrum indicated that the *m/z* 2332 peptide was a C-terminal fragment of PSA. This result suggested that the PSA fragment was composed of the following 19 amino acid residues: YTKVVHYRKWIKDTIVANP. The MALDI-DIT-TOF/MS^2^ mass spectrum of the synthesized peptide (Medical & Biological Laboratories Co., Ltd, Nagoya, Japan) coincided with that of the urinary *m/z* 2332 peptide (Figure 4b), which confirmed that both peptides consisted of the same amino acid sequence: YTKVVHYRKWIKDTIVANP. Moreover, its amino acid sequence, which was determined by Edman degradation, confirmed that it was a C-terminal fragment of PSA that consisted of 19 amino acid residues (data not shown). In the present study, we focused on urine samples voided following prostate massage, i.e., digital rectal examination (DRE). The origin of the peptide at *m/z* 2332 was investigated, and mass spectra were compared with the extracted fractions from urinary samples before and after DRE (Figure 5). The peak at *m/z* 2332 was exclusively detected after DRE. These results support 2331 Da peptide fragments being secreted from prostate glands by DRE.

### Quantitative analyses of a new potential pathognomonic biomarker using MALDI-DIT-TOF/MS

As was previously shown, the peak intensity at *m/z* 2332.3 was isolated from the S-plot as a potential biomarker candidate for distinguishing between the non-cancer and PCa groups. Thus, the urinary 2331 Da peptide was evaluated quantitatively using 50 urine samples voided from PCa patients (Table 1) and 19 from non-cancer subjects (Table 2) following prostate massage. A part of each data set had also been used in the discovery step.

**Figure 6:**
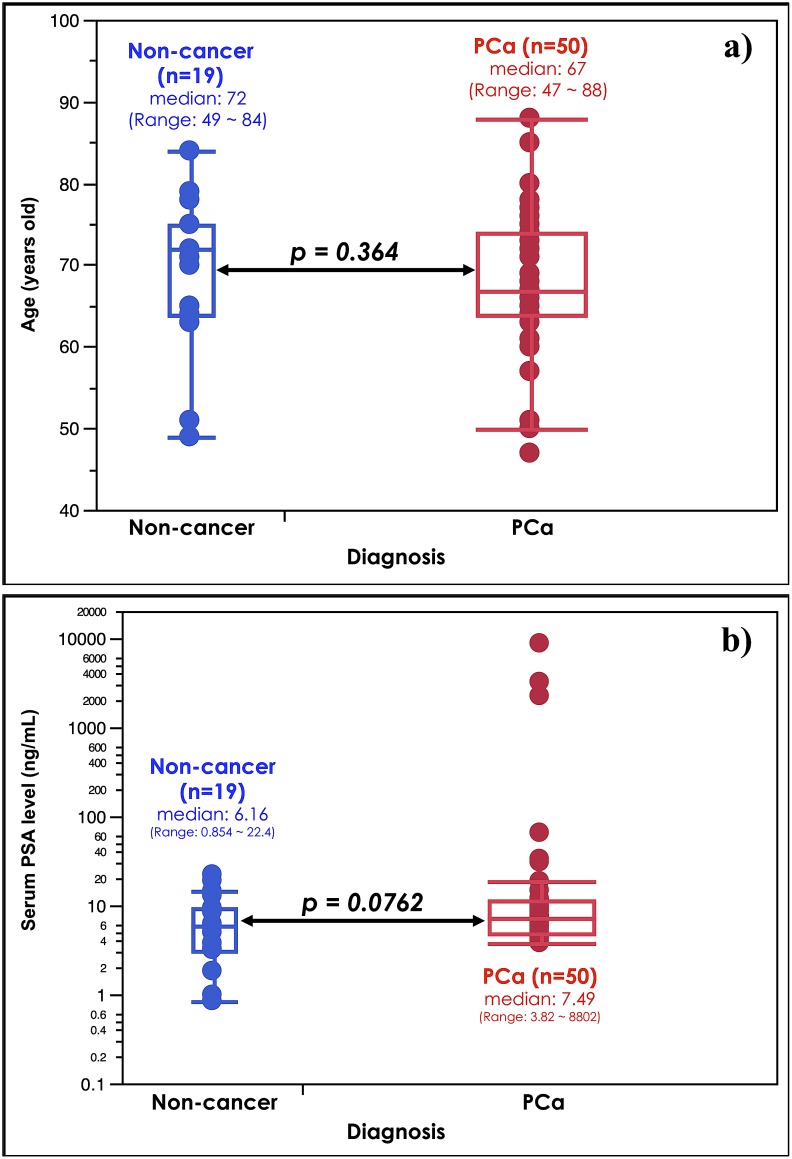
Clinical characteristics of non-cancer subjects and PCa patients used as a validation set. Age (a) and serum PSA (b) distributions between non-cancer subjects and PCa patients in the validation sets. No significant differences were observed in age or serum PSA between non-cancer subjects (n = 19) and PCa patients (n = 50). Their *p* values were 0.364 and 0.076, respectively.

The age and PSA distributions of all subjects are shown in Figure 6a and b. No significant differences were observed in age or PSA distribution between the non-cancer (n = 19) and PCa (n = 50) groups.

**Figure 7:**
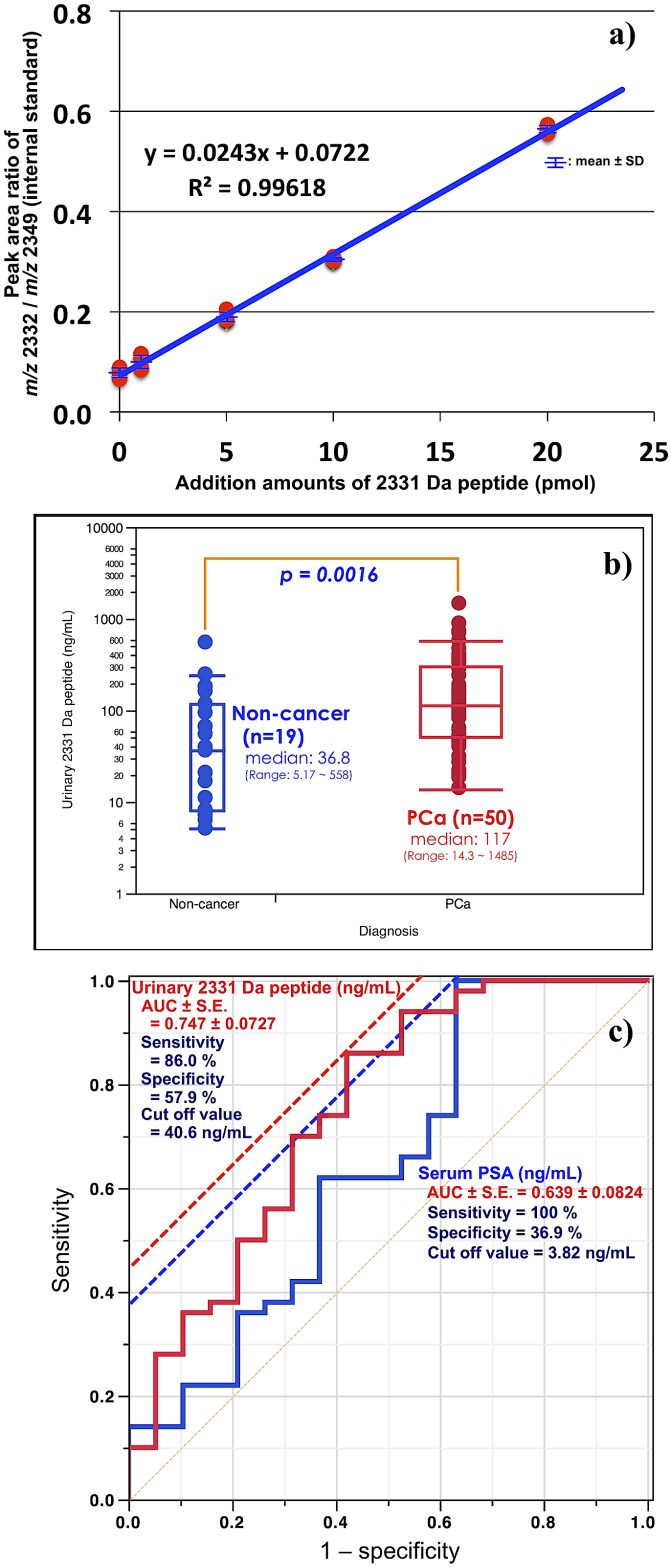
Quantitative analyses using a stable isotope-labeled internal standard. (a) Relationship between the peak area ratio between *m/z* 2332 and 2349 (stable isotope-labeled synthetic peptide; internal standard [iSTD]), and additional 2331 Da (non-labeled synthetic) peptide amounts. The additional contents of the iSTD were fixed in 20 pmol/1.0 mL urine. A standard curve regarding the additional amounts of the 2331 Da peptide is shown. Adequate correlations were observed in quantitative analyses of the 2331 Da peptide using MALDI-DIT-TOF/MS. (b) A comparison between pathological categories and urinary 2331 Da peptide concentrations. A significant difference (*p = 0.0016*) was observed between the non-cancer (n = 19) and PCa (n = 50) groups. (c) The ROC curves were calculated from the quantitative results of the two (i.e., non-cancer and PCa) groups. The AUCs of serum PSA level and urinary 2331 Da peptide concentration were 0.639 and 0.747, respectively. Regarding serum PSA, the cut-off level of 3.82 ng/mL had a sensitivity of 100% and specificity of 36.9%. Regarding urinary 2331 Da peptide, the cut-off level of 40.6 ng/mL had a sensitivity of 86.0% and specificity of 57.9%.

A representative urine sample analyzed with the addition of the internal standard (iSTD) was used to estimate the ratio between the peak areas at *m/z* 2332 for a non-labeled synthetic peptide and at *m/z* 2348 for a stable isotope-labeled synthetic peptide (YTKVVHY[R-^13^C_6_, -^15^N_4_]KWIKDT[I-^13^C_6_, -^15^N]VANP), respectively. The addition contents of the iSTD (stable isotope-labeled 2348 Da peptide amount) were fixed in 20 pmol/1.0 mL urine. A standard curve for additional amounts of the 2331 Da peptide is shown in Figure 7a. A strong correlation was observed between the peak area ratio between *m/z* 2332 and 2349 and the additional 2331 Da peptide amounts, with a regression equation of y = 0.0243x+0.0722 (x-axis: additional amounts of the 2331 Da peptide; y-axis; peak area ratio of *m/z* 2332/*m/z* 2349 [iSTD]; R^2^ = 0.996). A linear standard curve was obtained.

A significant difference was observed in urinary 2331 Da peptide concentrations between the non-cancer and PCa groups (Figure 7b). The medians of the 2331 Da peptide concentration in the non-cancer and PCa groups were 36.8 and 117 ng/mL, respectively. This result indicated that it might be possible to distinguish PCa patients from non-cancer subjects.

The ROC curves calculated from these results of two groups are shown in Figure 7c. Regarding serum PSA, an area under the curve (AUC) was 0.639. The cut-off level of 3.82 ng/mL had a sensitivity of 100% and specificity of 36.9%. On the other hand, an AUC was 0.747 regarding urinary 2331 Da peptide. The cut-off level of 40.6 ng/mL had a sensitivity of 86.0% and specificity of 57.9%.

No correlations were observed between the 2331 Da peptide concentration and serum PSA levels or PCa stages or the Gleason scores of patients (data not shown).

## Discussion

The diagnosis of cancer presents a major challenge for research and clinical management; extensive genomic, transcriptomic, and proteomic studies are being conducted in order to discover and identify biomarker candidates for use in high-throughput systems capable of large population screening [29]. A biomarker study is essential for understanding carcinogenesis and optimizing diagnostics and patient monitoring, and is also an important tool for the discovery and validation of new drugs. It addresses multiple classes of analyses and relies on both laboratory data and information technologies. Although a relatively novel research field, the discovery of biomarkers has the characteristics of a basic research approach that is closely related to system biology, and a clinical, translational shell application, in which validation and robustness are indispensable [30]. One of the important goals of cancer research is to discover biomarkers that are identifiable through simple, less invasive methods and have the potential to 1) identify the risk of cancer, 2) improve early detection, and 3) display utility in accurate staging and treatment monitoring [31].

PCa will affect one in five men and is now the second leading cause of cancer death among men in Western countries [1], [32]. Biomarkers play pivotal roles in the management of cancer patients. However, the biomarkers that are currently used for PCa are sub-optimal. For example, serum PSA, the most widely used biomarker, is controversial because of its sensitivity in various populations [33]. Concerns have also been expressed regarding the possible overdiagnosis of PCa by PSA in patients with limited potential for disease progression [34]–[36]. DRE is a common screening tool, in addition to serum PSA testing, for identifying patients at an increased risk of PCa. However, both tests are limited by low sensitivity and have led to many unnecessary biopsies [35]. None of those tests have proven to be useful for clinical testing; hence, several new biomarkers are currently being examined [37]–[42].

In the present study, we focused on urine voided following prostate massage, with the expectation that DRE urine samples contained various peptides and protein fragments secreted from the prostate gland, especially by prostate cancer cells. Urine samples were subjected to peptidomic and proteomic analyses using MALDI-DIT-TOF/MS. We were able to detect mass spectra differences in urine samples between non-cancer subjects and PCa patients. The most pronounced peaks were detected around *m/z* 2332.3 between the 2 groups. A new pathognomonic biomarker candidate around *m/z* 2332.3 was discovered in DRE urine samples obtained from PCa patients and analyzed by both MALDI-DIT-TOF/MS and multivariate analyses. The biomarker candidate was confirmed to be a 2331 Da peptide by the following procedures: 1) Mascot search engine using MS^2^ spectra concerning the *m/z* 2332 peak; 2) MS^2^ spectra comparisons between the synthesized *m/z* 2332 peptide according to the amino acid sequence assignment by the Mascot MS^2^ search and peptide extracted from the urine samples of PCa patients; 3) amino acid sequence analysis by Edman degradation; and 4) quantitative analyses of intact peptide concentrations using the stable isotope-labeled peptide. The 2331 Da peptide was identified as a C-terminal PSA fragment composed of 19 amino acid residues: YTKVVHYRKWIKDTIVANP. The results of the quantitative analyses regarding 2331 Da peptide concentrations in urinary extracts indicated that this peptide may be a new pathognomonic biomarker that can distinguish between non-cancer subjects and PCa patients.

Urine samples collected prior to HoLEP from non-cancer subjects were used in the multivariate analyses performed in the present study because the possibility of PCa in non-cancer subjects was negligible as most of these patients had negative prostate biopsy results before HoLEP. However, a PCa patient was detected in the HoLEP group (PCa No. 26 in Table 1), which demonstrated that it was very difficult to definitively deny the presence of PCa in non-cancer subjects, at least with recent clinical modalities.

Quantitative analyses were attempted for the potential pathognomonic biomarker using MALDI-DIT-TOF/MS. As shown in Figure 7a, a strong correlation was observed between the peak area ratio between *m/z* 2332 and 2349, and the additional 2331 Da peptide amounts. A linear standard curve (R^2^ = 0.996) was obtained. These results demonstrated that the quantitative analysis of the potential pathognomonic biomarker using MALDI-TOF/MS was simple, useful, and reproducible. These results are supported by previous observations described by Sogawa et al. [26] and Tyan et al. [27], in which the analyses with iSTDs were found to be simple and easy to apply to MALDI-TOF/MS analyses. As shown in Figure 7b, quantitative analyses revealed a significant difference in urinary 2331 Da peptide concentrations between the non-cancer and PCa groups. Therefore, the quantitative analyses of the 2331 Da peptide made it possible to distinguish PCa patients from non-cancer subjects.

The ROC curves were calculated from the quantitative results of two groups (Figure 7c). The AUCs of serum PSA level and urinary 2331 Da peptide concentration were 0.639 and 0.747, respectively. When diagnosing PCa by the urinary 2331 Da peptide concentration but not serum PSA level, the specificity was raised from 36.9% to 57.9% (a 1.57-fold increase), although the sensitivity reduced by 14%. Therefore, these results indicate that the diagnosis using the urinary peptide marker is superior in specificity for detecting PCa but sensitivity is inferior to the serum PSA. These results suggest that the biomarker concentration in the urine sample might be more useful than that of serum PSA for diagnosing PCa.

We performed a sequence search using the PMAP-CutDB proteolytic event database (URL: http://cutdb.burnham.org/) in order to detect candidate proteases that cleave specific amino acid sequences in PSA. CutDB is one of the PMAP web sites whose datasets are managed by the Center on Proteolytic Pathways at the Burnham Institute for Medical Research (La Jolla, CA, USA). The cleavage site identified in the present study was the RPSL-YTKV of the 2331 Da peptide. To the best of our knowledge, the cleavage site in the RPSL-YTKV sequence of the 2331 Da peptide has not been reported for PSA or KLK3. We proposed the following three hypotheses for the intracellular and/or extracellular PSA fragmentation mechanisms: 1) a new or mutant protease that acquired the neo-cleavage site(s) of the amino acid sequences was involved in fragmentation [43], [44]; 2) non-site-specific enzymatic degradation, which was caused by the co-localization of several proteases, was mutually related [45]; and 3) specific or non-specific protease activity was enhanced by one or several bio-factors that were overexpressed [46]–[48]. Based on the above hypotheses, we are continuing our research in order to elucidate the PSA fragmentation mechanism.

In conclusion, the results of the present study indicate that the 2331 Da peptide fragment of PSA may become a new pathognomonic biomarker for the diagnosis of PCa. A further large-scale investigation is currently underway to prove the applicability of this peptide in the early detection of PCa.
